# *Cordyceps cicadae* polysaccharide and plant extract as a potential antibiotic-free feed additive: effects on growth performance, antioxidant capacity, and cecal microbiota of Yandang chickens

**DOI:** 10.3389/fvets.2026.1878293

**Published:** 2026-06-26

**Authors:** Ruiping Li, Suzhen Liu, Liyan Dong, Zhangguo Liu, Shixiang Xu, Tao Zeng, Chong Li, He Ma, Yiqiu Chai

**Affiliations:** 1 Wenzhou Vocational College of Science and Technology, Wenzhou, China; 2 Zhejiang A&F University, Hangzhou, China; 3 Zhejiang Academy of Agricultural Sciences, Hangzhou, China; 4 Key Laboratory of Entomogenous Fungus Resources Research and Development of Wenzhou, Zhejiang Institute of Subtropical Crops, Wenzhou, China

**Keywords:** antibiotic-free production, antioxidant status, *cordyceps cicadae* polysaccharide, growth performance, gut microbiota, Yandang chicken

## Abstract

This study was conducted to investigate the effects of a composite of *Cordyceps cicadae polysaccharide* (CCP) and plant extract (PE) on growth performance, serum antioxidant status, and cecal microbiota of Yandang chickens, a Chinese indigenous breed, with the aim of evaluating its potential as a natural feed additive candidate for antibiotic-free poultry production. A total of 315 1-day-old healthy Yandang chickens were randomly allocated to seven groups: blank control, vaccinated control, antibiotic control, CCP group, PE group, low-dose combination group, and high-dose combination group. Supplementation with CCP at 50 mg/kg BW significantly increased average daily gain (ADG) compared with the vaccinated control (*p* < 0.05); the high-dose combination did not differ significantly from the CCP group in ADG. All additive-treated groups significantly elevated serum total protein and albumin levels while reducing creatinine content (*p* < 0.05). The CCP group and high-dose combination group significantly increased serum total antioxidant capacity (T-AOC), superoxide dismutase (SOD), catalase (CAT), and glutathione peroxidase (GSH-Px) activities, and markedly decreased malondialdehyde (MDA) content (*p* < 0.05), with the high-dose combination showing the strongest antioxidant activity among all treatment groups. Cecal microbiota analysis revealed that the high-dose combination significantly increased the relative abundance of Bacteroidetes, a phylum known for its capacity to degrade complex polysaccharides. PERMANOVA based on Bray–Curtis distance revealed significant differences in overall microbial community structure among groups (*p* = 0.001). In conclusion, CCP supplementation was associated with improved growth performance, while the CCP-PE composite, especially the high-dose combination, enhanced antioxidant capacity and modulated cecal microbiota in Yandang chickens under the conditions of this study. The high-dose combination exerted the optimal comprehensive effects on antioxidant capacity and intestinal flora, and may be considered a promising natural feed additive candidate for antibiotic-free poultry production.

## 1 Introduction

The global ban on in-feed antibiotics, driven by growing concerns over bacterial resistance, drug residues, and intestinal microbiota disturbance, has created an urgent need for safe, green, and residue-free alternatives in sustainable poultry production. Chinese herbal polysaccharides and plant extracts are considered promising natural feed additive candidates due to their antioxidant, immunomodulatory, and gut microbiota-modulating properties and leave no harmful drug residues after application (1).

*Cordyceps cicadae* polysaccharide (CCP) is a major bioactive component of the traditional entomopathogenic fungus Cordyceps cicadae. Our previous studies have demonstrated that CCP improves antioxidant capacity (2), enhances immune function (3), and improves growth performance (4) in chickens. Recent evidence has further shown that CCP can regulate signaling pathways such as NF-κB and MAPK and modulate gut microbiota metabolism (5).

Similarly, plant extracts (PE) have been widely reported to improve nutrient digestibility, intestinal morphology, and antioxidant status in poultry (6–8). However, most studies have focused on the individual effects of CCP or plant extracts alone, while the combined effects and co-application potential of CCP and PE remain largely unexplored. Given their functional complementarity-polysaccharides acting as prebiotic substrates to maintain gut microbial balance, and plant extracts providing potent antioxidant and anti-inflammatory activities-we hypothesize that their combined use may produce a more comprehensive protective effect than either component alone.

To test this hypothesis, the present study was conducted to investigate the effects of dietary supplementation with CCP, PE, and their combinations on growth performance, serum biochemical and antioxidant indices, and cecal microbiota community structure in Yandang chickens, a Chinese indigenous breed. This study aims to determine the optimal application scheme and provide a scientific basis for developing the CCP-PE composite as a novel green feed additive candidate for antibiotic-free poultry production.

## 2 Materials and methods

### 2.1 Ethics statement

The animal trial was conducted at the Wenzhou Seed and Seedling Science Park. All experimental procedures were approved by the Institutional Animal Care and Use Committee of the Zhejiang Academy of Agricultural Sciences (Approval No.: 2022ZAASLA59). All efforts were made to minimize animal suffering, and humane treatment was implemented during all procedures, including sample collection and euthanasia by cervical dislocation.

### 2.2 Experimental reagent

CCP was provided by the Zhejiang Institute of Subtropical Crops (Zhejiang, China). It was prepared as a light-yellow powder from the fruiting bodies of Cordyceps cicadae through drying, hot-water extraction, ethanol precipitation, deproteinization via the Sevag method, macroporous resin purification, and final drying, following established protocols.

A compound essential oil product (Guangdong Reborn Technology Group Co., Ltd., Guangdong, China) was used as the PE. This additive was a flowable, microencapsulated powder. According to the manufacturer's specification, its main active components were cinnamaldehyde (≥ 6%), carvacrol (≥ 0.2%), and eucalyptus oil (≥ 0.5%), formulated with mixed botanical extracts as the carrier (≥ 30%) derived from *Coptis chinensis, Cinnamomum cassia, Syzygium aromaticum*, and *Pogostemon cablin*.

Zinc bacitracin (Lukang Biochemistry Co., Ltd., batch no. P181013) was used as the antibiotic control. The live Newcastle disease vaccine (La Sota strain) was obtained from Bomeilai Biological Products Co., Ltd.

### 2.3 Experimental animals and management

A total of 315 one-day-old healthy Yandang chickens (half male and half female) with similar initial body weights were obtained from Zhejiang Lüyan Co., Ltd. (Zhejiang, China). The experiment was conducted at the Wenzhou Seed and Seedling Science Park. Birds were housed in a double-tiered cage system under natural lighting and ventilation. Routine sanitation procedures were strictly implemented throughout the 42-day experimental period.

Chickens were randomly allocated to seven treatment groups with three replicates per group and 15 birds per replicate. All birds had ad libitum access to feed and clean drinking water. A basal diet formulated to meet the nutritional requirements of Yandang chickens was provided by Wenzhou Zhengtai Agriculture and Animal Husbandry Co., Ltd.; its composition and nutritional levels are presented in Table 1.

**Table 1:** Ingredients and nutrient levels of the antibiotic-free basal diet (%, air-dry basis).

Ingredient	Content (1–3 weeks)	Content (4–6 weeks)
Corn	58.60	67.70
Wheat bran	8.35	5.10
Wheat middlings	6.00	6.00
Corn gluten meal	6.00	7.00
Soybean meal	16.95	10.1
Limestone	1.55	1.55
Premix^*^	2.55	2.55
Total	100.00	100.00
Nutrient Levels^#^		
Nutrient	Content (1–3 weeks)	Content (4–6 weeks)
Metabolizable energy (MJ/kg)	12.60	12.70
Crude protein	21.27	19.94
Calcium	1.01	0.85
Total phosphorus	0.6	0.54
Methionine	0.50	0.40
Lysine	1.15	1.10

^*^The premix provided per kilogram of diet: Vitamin A 5,000 IU, Vitamin B_1_ 9.8 mg, Vitamin B_2_ 28.8 mg, Vitamin B_6_ 19.6 mg, Vitamin B_12_ 0.1 mg, Vitamin D 10,000 IU, Vitamin E 75.0 mg, Vitamin K_3_ 18.8 mg, nicotinic acid 196.5 mg, folic acid 4.8 mg, biotin 2.5 mg, Cu (as copper sulfate) 4.0 mg, Fe (as ferrous sulfate) 40.0 mg, Zn (as zinc sulfate) 37.6 mg, Mn (as manganese sulfate) 50.0 mg, Se (as sodium selenite) 0.2 mg, I (as potassium iodide) 0.2 mg.

^#^Nutrient levels were calculated values.

Except for the blank control group (Group I), all birds were vaccinated against Newcastle disease via eye drop with a live attenuated vaccine (La Sota strain) on days 14 and 28. Health status and general behavior were observed and recorded daily. The blank control was intentionally maintained without vaccination to serve as a non-immunized baseline reference, to assess the potential growth depression associated with vaccination.

### 2.4 Experimental design

A single-factor completely randomized design was adopted. A total of 315 Yandang chickens were randomly assigned to seven treatment groups, with three replicates of 15 birds each. The treatment groups were as follows: blank control (Group I), vaccinated control (Group II), antibiotic control (Group III), CCP alone at 50 mg/kg BW (Group IV), low-dose combination of CCP 25 mg/kg BW + PE 125 mg/kg BW (Group V), high-dose combination of CCP 50 mg/kg BW + PE 125 mg/kg BW (Group VI), and PE alone at 250 mg/kg BW (Group VII). Detailed treatment information is presented in Table 2.

**Table 2:** Experimental design and treatment details.

Group	Treatment description	Additive and dosage (administered in 0.5 mL solution per bird)	Vaccination status
I	Blank control	Physiological saline (0 mg/kg BW)	No
II	Vaccinated control	Physiological saline (0 mg/kg BW)	Yes^*^
III	Antibiotic control	Zinc bacitracin (50 mg/kg BW)	Yes^*^
IV	*Cordyceps cicadae* polysaccharide	CCP (50 mg/kg BW)	Yes^*^
V	Low-dose combination	CCP (25 mg/kg BW) + PE (125 mg/kg BW)	Yes^*^
VI	High-dose combination	CCP (50 mg/kg BW) + PE (125 mg/kg BW)	Yes^*^
VII	Plant extract	PE (250 mg/kg BW)	Yes^*^

^*^Birds in Groups II to VII were vaccinated against Newcastle disease on days 14 and 28.

BW, body weight; CCP, Cordyceps cicadae polysaccharide. The PE refers to the compound essential oil product described in Section 2.2.

All additives were administered via oral gavage (0.5 mL per bird) once daily at 14:00 during two periods: days 11–17 and days 25–31. This route was selected to ensure precise and uniform intake of the test substances by each individual bird. All groups, including the blank and vaccinated controls, received the same gavage and eye-drop procedures with physiological saline to eliminate any potential confounding effects from handling stress.

### 2.5 Measurements and analytical methods

#### 2.5.1 Determination of growth performance

On days 10 and 42 of the experiment, all chickens were fasted for 12 h, and their individual fasting body weights were accurately recorded. Feed intake per replicate was precisely determined by recording the amount of feed offered and the amount refused throughout the experimental period. ADG, ADFI, and F/G were calculated for each treatment group.

#### 2.5.2 Determination of serum biochemical and antioxidant indices

On day 42, six birds were randomly selected from each replicate (18 birds per treatment group) for blood collection from the wing vein. Blood samples were left to clot at ambient temperature before centrifugation at 3,000 r/min for 15 min at 4 °C to separate the serum. The serum samples were stored at −20 °C until analysis.

Serum concentrations of TP, TC, ALB, TG, CREA, and UN were determined using colorimetric kits (Beijing Huaying Biotechnology Research Institute, Beijing, China) on a Mindray BS-420 automatic biochemical analyzer. Serum T-AOC, as well as the activities of SOD, GSH-Px, CAT, and the content of MDA, were measured using corresponding colorimetric kits from the same manufacturer on an A6 semi-automatic biochemical analyzer (Beijing Songshang Technology Co., Ltd., Beijing, China), following the manufacturer's instructions.

#### 2.5.3 Analysis of cecal microbial diversity

On day 42, three birds were randomly selected from each replicate (nine birds per treatment group) and euthanized by cervical dislocation. The cecal contents were aseptically collected into sterile cryopreservation tubes, immediately frozen in liquid nitrogen, and stored at −80 °C until DNA extraction.

Total bacterial genomic DNA was extracted from the cecal content samples using the PowerSoil^®^ DNA Isolation Kit (MO BIO Laboratories, Carlsbad, CA, USA) according to the manufacturer's protocol. The concentration and purity of the extracted DNA were determined by A260/A280 ratio, and DNA integrity was verified by 1.8% agarose gel electrophoresis.

The V3-V4 hypervariable region of the bacterial 16S rRNA gene was amplified by PCR using the universal primer pair 338F (5′-ACTCCTACGGGAGGCAGCA3′) and 806R (5′-GGACTACHVGGGTWTCTAAT3′). PCR amplification was performed using high-fidelity DNA polymerase (Q5, New England Biolabs). The PCR products were purified using VAHTSTM DNA Clean Beads, quantified using Quant-iTTM dsDNA HS reagent, and pooled in equal amounts. Sequencing libraries were constructed using the TruSeq^®^ DNA PCR-Free Sample Preparation Kit (Illumina, San Diego, CA, USA), and library quality was assessed using a Qubit fluorometer (Thermo Fisher Scientific, Waltham, MA, USA). Paired-end sequencing (PE250) was performed on the Illumina HiSeq 2,500 platform by Beijing Biomarker Technologies Co., Ltd. (Beijing, China).

Raw sequencing reads were subjected to quality control, filtering, and assembly to generate high-quality clean tags. Operational taxonomic units (OTUs) were clustered at 97% sequence similarity using USEARCH software (9). Taxonomic assignment of OTUs was performed against the Silva bacterial taxonomic database (Release 132). Alpha diversity indices (Chao1, Ace, Shannon, and Simpson) and beta diversity (PCoA) were calculated and visualized using the QIIME (version 1.9.1) pipeline and R software v3.6.3 (The R Foundation for Statistical Computing, Vienna, Austria).

#### 2.5.4 Statistical analysis

All experimental data were analyzed using SPSS version 26.0 (IBM Corp., Armonk, NY, USA). For growth performance parameters (ADG, ADFI, and F/G), cage means served as the experimental unit (*n* = 3 per group). For serum biochemical, antioxidant, and cecal microbiota indices, individual birds served as the statistical unit. Differences among groups were examined by one-way ANOVA followed by the Student–Newman–Keuls (S–N–K) test for multiple comparisons. Unless otherwise stated, the vaccinated control (Group II) served as the primary comparator for treatment effects. Statistical significance was set at *p* < 0.05. All results are presented as mean ± SD.

## 3 Results

### 3.1 Growth performance and serum biochemical indices

As shown in Table 3, ADG was significantly higher in Groups I and IV compared with Group II (*p* < 0.05). ADFI was significantly higher in Groups I, IV, and VI compared with Group II (*p* < 0.05). Feed-to-gain ratio (F/G) in all treatment groups (IV–VII) was numerically lower than in the vaccinated control (Group II), although the differences were not significant (*p* > 0.05). Group III also showed no significant difference in F/G compared with Group II (*p* > 0.05).

**Table 3:** Growth performance of Yandang chickens.

Group	ADG (g)	ADFI (g/d)	F/G
I	10.47 ± 1.58^a^	26.10 ± 0.63^a^	2.49 ± 0.18
II	9.21 ± 1.41^c^	23.30 ± 0.54^f^	2.53 ± 0.08
III	9.56 ± 1.24^abc^	23.90 ± 0.59^d^	2.50 ± 0.24
IV	10.23 ± 1.44^ab^	24.35 ± 0.41^c^	2.38 ± 0.10
V	9.75 ± 1.63^abc^	23.69 ± 0.32^e^	2.43 ± 0.14
VI	10.06 ± 1.63^abc^	24.85 ± 0.34^b^	2.47 ± 0.14
VII	9.49 ± 1.76^bc^	23.44 ± 0.71^f^	2.47 ± 0.03

In each column, means marked with different lowercase letters differ significantly at *p* < 0.05; this notation applies to all subsequent tables.

Serum biochemical parameters are presented in Table 4. Serum TP and ALB levels in all treatment groups (IV–VII) were significantly higher than those in Group II (*p* < 0.05). Serum CREA content in all treatment groups (IV–VII) was significantly lower than in the vaccinated Groups II (*p* < 0.05). Serum TC was significantly higher in Groups III, IV, V, VI, and VII compared with Group II (*p* < 0.05). Serum UN in Group VI and TG in Group V were numerically lower than in their respective control groups; however, these differences did not reach statistical significance (*p* > 0.05).

**Table 4:** Serum biochemical parameters of Yandang chickens.

Group	TP (g/L)	TC (mmol/L)	ALB (g/L)	CREA (μmol/L)	UN (mg/dl)	TG (mmol/L)
I	18.87 ± 7.17^b^	1.89 ± 0.70^bc^	9.05 ± 3.44^b^	28.34 ± 8.25^a^	1.26 ± 0.40	0.37 ± 0.08
II	16.07 ± 4.32^c^	1.57 ± 0.67^c^	7.91 ± 2.47^b^	29.89 ± 8.28^a^	1.24 ± 0.19	0.32 ± 0.11
III	25.22 ± 2.30^a^	2.30 ± 0.37^ab^	11.56 ± 1.02^a^	23.26 ± 2.87^b^	1.26 ± 0.15	0.38 ± 0.07
IV	23.28 ± 4.85^a^	2.01 ± 0.51^ab^	10.35 ± 1.60^a^	22.54 ± 3.25^b^	1.28 ± 0.18	0.33 ± 0.08
V	23.66 ± 3.58^a^	2.01 ± 0.31^ab^	10.68 ± 1.45^a^	22.49 ± 2.45^b^	1.24 ± 0.18	0.31 ± 0.08
VI	23.95 ± 0.15^a^	2.17 ± 0.22^ab^	11.08 ± 1.11^a^	21.64 ± 2.59^b^	1.13 ± 0.11	0.33 ± 0.06
VII	25.47 ± 2.15^a^	2.38 ± 0.31^a^	11.58 ± 0.98^a^	23.06 ± 2.25^b^	1.21 ± 0.18	0.35 ± 0.08

Different lowercase superscript letters (a, b, c) within the same column represent significant differences between groups at *p* < 0.05; values marked with identical letters have no statistically significant difference.

### 3.2 Serum antioxidant indices

As shown in Table 5, Group VI exhibited the highest T-AOC among all groups (*p* < 0.05), followed by Group IV, which was significantly higher than all groups except Group VI (*p* < 0.05). For SOD, CAT, and GSH-Px, both Groups IV and VI exhibited significantly higher activities compared with Groups I, II, and III (*p* < 0.05).

**Table 5:** Serum antioxidant indices of Yandang chickens.

Group	T-AOC (U/mL)	SOD (U/mL)	CAT (U/mL)	GSH-Px (U/mL)	MDA (nmol/mL)
I	11.03 ± 0.82^d^	71.42 ± 3.10^d^	46.95 ± 4.10^d^	833.07 ± 31.52^d^	4.74 ± 0.55^c^
II	12.98 ± 1.20^c^	79.14 ± 3.52^c^	52.05 ± 5.24^c^	875.88 ± 19.75^c^	4.40 ± 0.35^d^
III	7.04 ± 1.09^f^	51.09 ± 1.72^g^	29.50 ± 2.52^g^	685.31 ± 10.53^g^	6.04 ± 0.45^a^
IV	14.78 ± 1.34^b^	85.71 ± 4.61^b^	58.37 ± 3.81^b^	919.78 ± 41.99^b^	4.06 ± 0.20^e^
V	10.44 ± 0.76^d^	65.90 ± 3.33e	41.56 ± 9.96^e^	811.45 ± 21.25^e^	5.26 ± 0.49^b^
VI	17.11 ± 1.35^a^	129.30 ± 5.19^a^	65.08 ± 5.01^a^	964.54 ± 22.87^a^	3.12 ± 0.48^f^
VII	9.39 ± 1.82^e^	60.25 ± 2.61^f^	33.49 ± 4.84^f^	755.64 ± 26.93^f^	5.53 ± 0.31^b^

Lowercase letters a and b indicate significant inter-group differences at *p* < 0.05; means sharing the same letter are not significantly different.

Notably, Groups I and II also showed significantly higher T-AOC, SOD, CAT, and GSH-Px activities, and lower MDA content, than Group III (*p* < 0.05).

### 3.3 Effects on the gut microbiota of Yandang chickens

#### 3.3.1 Sequencing results

**Figure 1:**
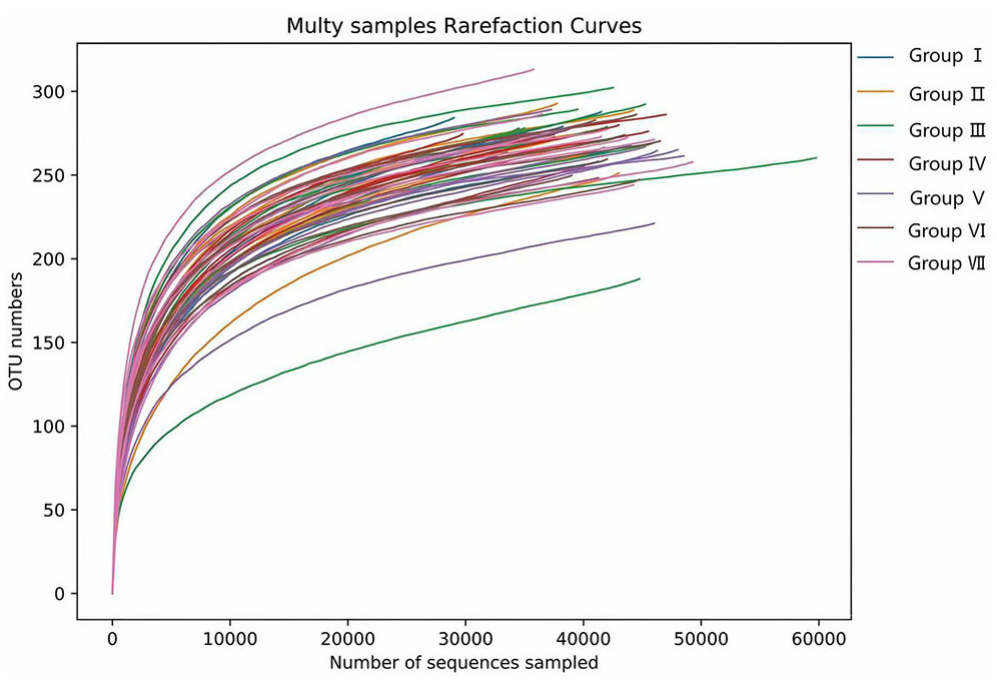
Rarefaction curves for cecal microbial samples of Yandang chickens. Each curve reflects the relationship between the number of sampled sequences and observed operational taxonomic units (OTUs) clustered at a 97% sequence similarity threshold. All curves gradually reach a plateau, confirming sufficient sequencing depth for microbiota analysis. Groups I–VII are defined in Table 2.

One sample from Group IV was unavailable for sequencing; therefore, a total of 62 cecal content samples were analyzed. From these samples, 4,799,221 paired-end reads were obtained. After assembly and quality filtering, 4,561,941 clean tags were generated, with a minimum of 47,773 and an average of 73,580 clean tags per sample. Rarefaction curves based on OTU numbers approached the saturation plateau (Figure 1), indicating that the sequencing depth was sufficient to capture the majority of microbial diversity present in the samples (10).

#### 3.3.2 Alpha diversity of gut microbiota

As shown in Table 6, Group VII exhibited a numerically lower Simpson index and a numerically higher Shannon index compared with Groups I–III, although the differences were not statistically significant (*p* > 0.05). These results suggest that PE supplementation may tend to increase the richness and evenness of the cecal microbiota.

**Table 6:** Gut microbial alpha diversity in Yandang chickens.

Group	Chao1	ACE	Simpson	Shannon
I	296.67 ± 19.54	294.73 ± 17.66	0.09 ± 0.04	3.34 ± 0.28^ab^
II	302.17 ± 12.37	296.81 ± 9.37	0.08 ± 0.04	3.36 ± 0.33^ab^
III	296.75 ± 19.21	297.77 ± 11.02	0.08 ± 0.04	3.44 ± 0.32^ab^
IV	302.01 ± 9.23	296.96 ± 8.33	0.09 ± 0.04	3.30 ± 0.24^ab^
V	294.76 ± 24.44	288.85 ± 20.30	0.11 ± 0.04	3.18 ± 0.36^b^
VI	296.98 ± 24.35	286.58 ± 15.06	0.09 ± 0.03	3.35 ± 0.25^ab^
VII	297.32 ± 16.57	292.62 ± 18.73	0.07 ± 0.04	3.56 ± 0.46^a^

Lowercase letters a and b indicate significant inter-group differences at *p* < 0.05; means sharing the same letter are not significantly different.

#### 3.3.3 Beta diversity of gut microbiota

**Figure 2:**
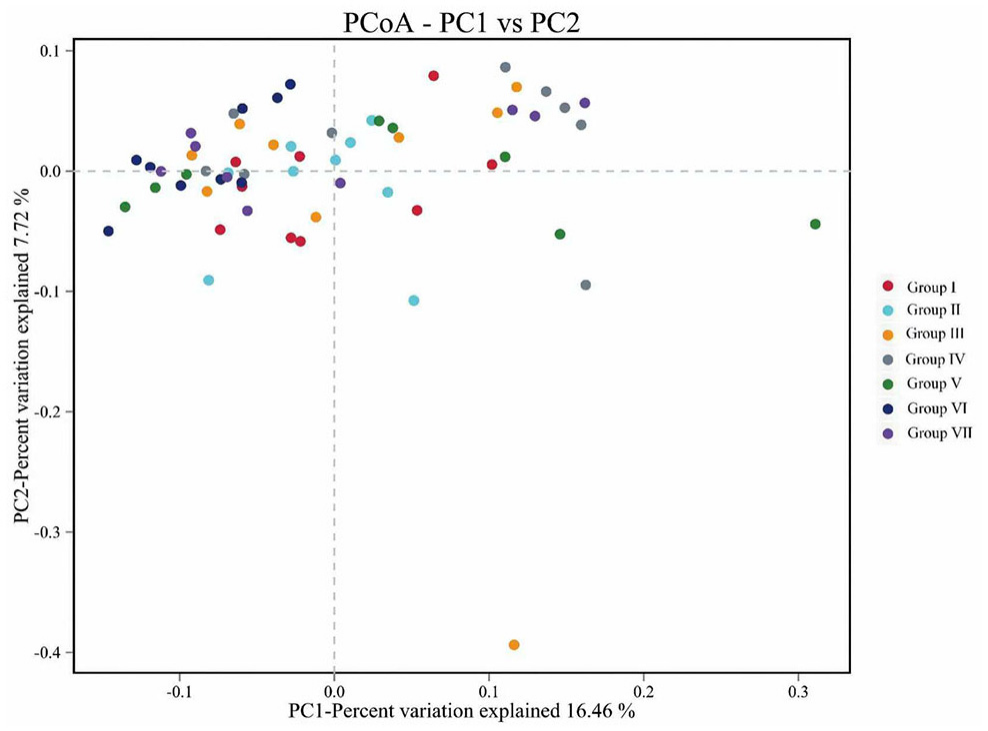
Principal coordinates analysis (PCoA) of cecal bacterial communities from Yandang chickens based on the Bray–Curtis distance matrix. Each dot represents an individual sample (*n* = 62). The percentage of total community variation explained by PC1 and PC2 is marked on the corresponding axes. Groups I–VII are defined in Table 2.

Principal coordinates analysis (PCoA) showed that samples from Groups II, IV, VI, and VII were relatively clustered, indicating high within-group similarity, whereas those from Group III were more dispersed (Figure 2). PERMANOVA based on Bray–Curtis distance revealed significant differences in overall microbial community structure among groups (*R*^2^ = 0.158, *p* = 0.001; Table 7).

**Table 7:** PERMANOVA results based on Bray—Curtis distance.

Source	df	Sum of squares	*R* ^2^	*F*	*p*-value
Group	6	0.528	0.158	1.718	0.001
Residual	55	2.815	0.842	—	—
Total	61	3.342	1.000	—	—

#### 3.3.4 Gut microbiota composition at the phylum level

**Figure 3:**
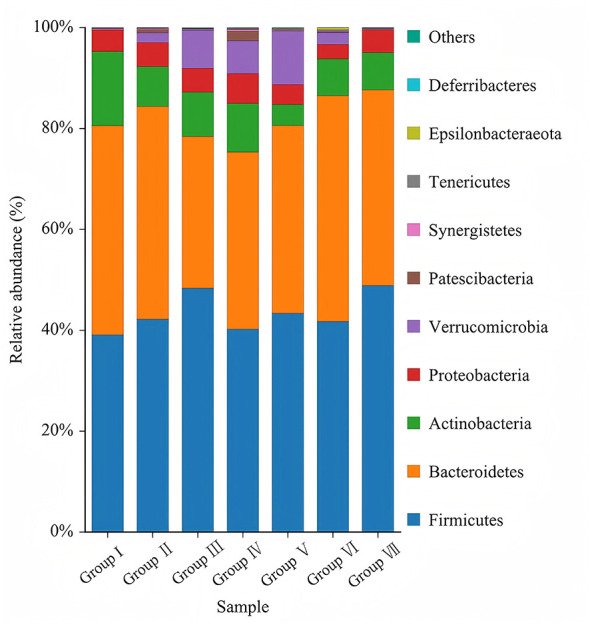
Relative abundance of cecal microbiota at the phylum level across experimental groups of Yandang chickens. Firmicutes and Bacteroidetes are the dominant phyla in the cecal ecosystem. Values are presented as the mean relative abundance (%) per treatment. Groups I–VII are defined in Table 2.

At the phylum level, Firmicutes and Bacteroidetes were the dominant phyla in the cecum, accounting for 39%−49% and 30%−45% of all sequences, respectively (Figure 3). Compared with Groups I–III, Group VII showed a 20.0%, 15.8%, and 1.1% increase in the relative abundance of Firmicutes, respectively. Group V also exhibited higher Firmicutes abundance than Groups I and II.

Regarding Bacteroidetes, Group VI showed a 7.4%, 5.8%, and 32.8% higher relative abundance compared with Groups I–III, respectively. Group VII also exhibited a 22.4% higher Bacteroidetes abundance than Group III.

## 4 Discussion

### 4.1 Effects on growth performance and protein and lipid metabolism

The significant increase in ADG observed in the CCP group (Group IV: CCP 50 mg/kg BW) compared with the vaccinated control (Group II) indicates that CCP alone was effective in promoting growth. The numerically highest ADG was observed in the blank control group (Group I), which is consistent with the expected absence of vaccination-induced immune stress, a physiological state known to divert nutrients away from growth. Although the high-dose combination group (Group VI) did not show a statistically significant increase in ADG relative to Group II, its ADG did not differ significantly from that of Group IV, suggesting that the addition of PE did not diminish the growth-promoting effect of CCP. This combined effect may be attributed to their complementary biological functions in the intestinal tract. Plant-derived active components, such as cinnamaldehyde and carvacrol, have been widely reported to improve nutrient utilization and digestive efficiency in poultry (11, 12) and can upregulate intestinal nutrient transporter genes and enhance digestive enzyme activities, thereby improving nutrient absorption (13). Meanwhile, as a prebiotic substrate, CCP can be fermented by gut microbiota to produce short-chain fatty acids (SCFAs), these metabolites presumably supply energy for intestinal epithelial cells and help maintain intestinal barrier integrity (14).

The additives were administered via oral gavage in this study to ensure precise and uniform intake of the test substances. For large-scale production, these dose levels can be used as a reference for in-feed inclusion via uniform mixing, with the gavage-derived responses providing a basis for designing feeding regimens.

All treatment groups (IV–VII) exhibited numerically lower F/G ratios than the vaccinated control (Group II), though the difference was not statistically significant, which is consistent with the above mechanistic interpretation. The lack of significant differences may be associated with the genetic characteristics of Yandang chickens, the relatively short 42-day experimental period, or insufficient dosage optimization. This interpretation is consistent with previous reports that the efficacy of plant essential oils is regulated by multiple interacting factors (15). Improved growth performance was accompanied by favorable changes in serum protein parameters. Compared with Group II, all treatment groups (IV–VII) had significantly higher serum TP and ALB levels and lower CREA content. Higher TP and ALB levels are consistent with improved protein metabolism, as reported for bioactive polysaccharides previously (16). The reduced serum CREA suggests more efficient protein turnover and decreased nitrogen excretion, which may be closely related to the improvement of intestinal microbial composition induced by the CCP-PE composite (17).

Serum TC was significantly higher in the additive-treated groups (Groups IV–VII) compared with Group II (*p* < 0.05), which may reflect enhanced lipid metabolism associated with CCP and PE supplementation. Serum UN and TG showed no significant differences among groups. The lower UN level in Group VI suggested higher amino acid utilization efficiency, while the decreased TG tendency in Group V may reflect enhanced lipid oxidation. Plant extracts are known to regulate lipid metabolism by modulating key metabolic enzymes and signaling pathways (17, 18).

Collectively, the increased serum TP and ALB, decreased CREA, and favorable trends in lipid metabolites were associated with improved growth performance and favorable changes in serum protein and lipid parameters. These findings provide a rationale for further evaluation of this compound as a potential feed additive candidate for antibiotic-free poultry production (19).

### 4.2 Effects on serum antioxidant capacity

The present study revealed two notable findings regarding systemic antioxidant status. First, Groups IV and VI exhibited enhanced systemic antioxidant defense compared with Groups II and III, as evidenced by elevated antioxidant enzyme activities and reduced MDA content. Notably, Group VI outperformed Group IV in T-AOC. Second, Groups I and II showed higher antioxidant activities and lower MDA levels than Group III, suggesting that zinc bacitracin may have suppressed endogenous antioxidant function (20, 21). This contrast highlights a physiological advantage of phytogenic additives over antibiotics in protecting against cellular oxidative injury.

The superior antioxidant profile observed in Group VI suggests a combined beneficial effect of CCP and PE. On one hand, CCP has been reported to activate the Nrf2-Keap1 signaling pathway, which may contribute to the upregulation of downstream antioxidant enzymes such as SOD, CAT, and GSH-Px (22, 23), although this pathway was not directly assessed in the present study. On the other hand, bioactive components in PE, including cinnamaldehyde and carvacrol, have been reported to possess direct free radical-scavenging capacities and may enhance cellular resistance to oxidative stress (13, 17). The combination of these distinct yet complementary mechanisms may provide a more comprehensive antioxidant defense than either component alone.

Collectively, the CCP-PE composite was associated with enhanced antioxidant capacity, as evidenced by increased antioxidant enzyme activities and reduced lipid peroxidation. Furthermore, the composite showed favorable redox-modulating effects compared with the antibiotic control, supporting its further evaluation as a candidate for antibiotic-free poultry production (19, 21).

### 4.3 Effects on the gut microbiota

The gut microbiota plays a critical role in maintaining host health, regulating nutrient metabolism, and modulating immune function (24, 25). In the present study, 16S rRNA high-throughput sequencing was used to evaluate the effects of CCP and PE on cecal microbial community structure in Yandang chickens. Changes in microbial composition and diversity may help explain the observed improvements in growth performance and antioxidant capacity from a gut microecological perspective.

#### 4.3.1 Analysis of gut microbial diversity

For alpha diversity, Group VII showed a numerically higher Shannon index and a numerically lower Simpson index compared with Groups I, II, and III, although these differences were not statistically significant (*p* > 0.05). This trend suggests that PE at 250 mg/kg BW may contribute to higher richness and evenness of the cecal microbial community (26).

PERMANOVA revealed significant differences in overall microbial community structure among groups. The relatively low *R*^2^ value suggests that treatment effects accounted for a limited proportion of the total variation. Samples from Group III were more dispersed than those from other groups, indicating lower stability and higher inter-individual variation in microbial community structure. These results are consistent with previous findings that antibiotic treatment can disrupt gut microbiota homeostasis and increase inter-individual variability (27, 28).

#### 4.3.2 Changes in gut microbiota composition

At the phylum level, Firmicutes and Bacteroidetes were the dominant constituents of the cecal microbiota, collectively accounting for more than 70% of the total microbial community (29, 30). Notably, the high-dose combination group (Group VI) exhibited a markedly higher relative abundance of Bacteroidetes compared with the control groups (Groups I–III), and Group VII also showed an increase relative to Group III. For Firmicutes, Group VII displayed a higher abundance relative to Groups I–III, and Group V showed an increase compared with Groups I and II.

The enrichment of Bacteroidetes in Group VI is of particular interest. As a major phylum specialized in the degradation of complex polysaccharides, Bacteroidetes possesses extensive enzymatic machinery to ferment dietary and host-derived glycans, producing SCFAs as primary metabolic end-products (31). These SCFAs—particularly acetate, propionate, and butyrate—are reported to serve as essential energy substrates for intestinal epithelial cells and contribute to maintaining gut barrier integrity, regulating host metabolism, and modulating systemic immune responses (32–35). The elevated Bacteroidetes abundance raises the plausible possibility that the CCP-PE blend improves intestinal carbohydrate fermentation capacity of the gut microbiota, providing a potential microecological explanation for the observed improvements in growth performance and systemic antioxidant capacity in Group VI.

#### 4.3.3 Potential links between gut microbiota remodeling and improved host physiological status

The present study showed that the high-dose combination of CCP and PE was associated with both improved growth performance and systemic antioxidant capacity, alongside an increased relative abundance of Bacteroidetes in the cecal microbiota. While these associations do not establish causality, they raise the possibility of a gut microbiota-mediated signaling axis through which the CCP-PE composite may contribute to the observed phenotypic improvements.

Bacteroidetes is a major phylum specialized in the degradation of complex polysaccharides, fermenting dietary and host-derived glycans into SCFAs—predominantly acetate, propionate, and butyrate (31, 34). These SCFAs serve as energy substrates for intestinal epithelial cells, help maintain gut barrier integrity, and can enter the portal circulation to reach distal organs including the liver and muscle (35). Through pathways such as the gut-liver axis, SCFAs can activate host cellular defense mechanisms, including the Nrf2-Keap1 antioxidant signaling pathway (22), which in turn upregulates the expression of antioxidant enzymes such as SOD, CAT, and GSH-Px while suppressing lipid peroxidation. This microbiota-metabolism-host axis, well-documented in the literature (21, 36), provides a plausible mechanistic framework linking the CCP-PE-induced enrichment of Bacteroidetes with the systemic benefits observed in Group VI.

Several limitations of the present study should be considered when interpreting these findings. First, SCFA concentrations were not directly measured in cecal contents or serum; the link between Bacteroidetes enrichment and enhanced SCFA production is therefore inferred from established microbial physiology. Second, although PERMANOVA revealed statistically significant differences in overall microbial community structure, the *R*^2^ value was low, suggesting that treatment effects accounted for a limited proportion of the total variation. This may reflect the limited sample size for sequencing or the 42-day trial duration (37). Future studies incorporating targeted SCFA metabolomics and direct assessment of intestinal barrier function and Nrf2 pathway activation are warranted to validate the proposed microbiota-mediatedmechanism.

## 5 Conclusions

This study showed that dietary supplementation with CCP was associated with improved growth performance, while the CCP-PE composite, especially the high-dose combination, enhanced antioxidant capacity and modulated cecal microbiota in Yandang chickens under the conditions of this study.

Supplementation with CCP at 50 mg/kg BW (Group IV) significantly increased ADG, while the high-dose combination (Group VI) did not differ significantly from Group IV in ADG. Both Group IV and Group VI significantly elevated serum TP and ALB levels and reduced CREA content, findings consistent with improved protein metabolism. Group VI exhibited the most pronounced antioxidant effects, with significantly higher serum T-AOC, SOD, CAT, and GSH-Px activities and lower MDA content compared with all other groups (*p* < 0.05), suggesting alleviation of oxidative stress.

Furthermore, the high-dose combination was associated with an increased relative abundance of Bacteroidetes in the cecum, a phylum known for its capacity to degrade complex polysaccharides.

Collectively, the CCP-PE composite, especially the high-dose combination, exhibited combined beneficial effects on antioxidant status and gut microbiota composition. These findings suggest that the CCP-PE composite may be considered a promising natural feed additive candidate for antibiotic-free poultry production. Further studies incorporating direct SCFA measurement, intestinal barrier assessment, and in-feed supplementation trials are warranted to validate these observations.

## Data Availability

The 16S rRNA gene sequencing data have been deposited in the NCBI Sequence Read Archive (SRA) under BioProject accession number PRJNA1464183. The other datasets generated and analyzed during this study are available from the corresponding author upon reasonable request.

## References

[1] Li X Jiao T Xu J Zhao S Liu J. Astragalus polysaccharide and *Agaricus Blazei* polysaccharides improve growth performance, meat quality and health status of lambs via rescheduling the rumen microbiota. Anim Feed Sci Technol. (2026) 334:116658. doi: 10.1016/j.anifeedsci.2026.116658

[2] Li R Liu Z Zhang S Dong L Wang Q Xu S . Effects of *Cordyceps cicadae* polysaccharide on growth performance, antioxidant capacity and intestinal microbial diversity of broiler chickens. Chin J Vet Sci. (2021) 41:1118–26. doi: 10.16303/j.cnki.1005-4545.2021.06.13

[3] Li R Liu Z Zhang S Dong L Wang Q Xu S . Effects of *Cordyceps cicadae* polysaccharide on lipid metabolism and immune function of chicken. Chin J Vet Sci. (2021) 41:1598–603. doi: 10.16303/j.cnki.1005-4545.2021.08.25

[4] Li R Liu Z Dong L Wang Q Xu S Li G . Effects of *Cordyceps cicadae* polysaccharide and plant extract on growth performance and immune function of Yandang ma-chicken. Chin J Anim Nutr. (2020) 32:5433–40. doi: 10.3969/j.issn.1006-267x.2020.11.050

[5] Sun L Yuan H Ma H Wang Y. Effects of *Cordyceps cicadae* polysaccharide on gut microbiota, the intestinal mucosal barrier, and inflammation in diabetic mice. Metabolites. (2025) 15:8. doi: 10.3390/metabo15010008 39852351 PMC11768040

[6] Brenes A Roura E. Essential oils in poultry nutrition: main effects and modes of action. Anim Feed Sci Technol. (2010) 158:1–14. doi: 10.1016/j.anifeedsci.2010.03.007

[7] Chowdhury S Mandal GP Patra AK Kumar P Samanta I Pradhan S . Different essential oils in diets of broiler chickens: 2. Gut microbes and morphology, immune response, and some blood profile and antioxidant enzymes. Anim Feed Sci Technol. (2017) 236:39–47. doi: 10.1016/j.anifeedsci.2017.12.003

[8] Chowdhury S Mandal GP Patra AK. Different essential oils in diets of chickens: 1. Growth performance, nutrient utilisation, nitrogen excretion, carcass traits and chemical composition of meat. Anim Feed Sci Technol. (2018) 236:86–97. doi: 10.1016/j.anifeedsci.2017.12.002

[9] Edgar RC. UPARSE: highly accurate OTU sequences from microbial amplicon reads. Nat Methods. (2013) 10:996–98. doi: 10.1038/nmeth.2604 23955772

[10] Wang Y Sheng HF He Y Wu JY Jiang YX Tam NF . Comparison of the levels of bacterial diversity in freshwater, intertidal wetland, and marine sediments by using millions of illumina tags. Appl Environ Microbiol. (2012) 78:8264–71. doi: 10.1128/AEM.01821-12 23001654 PMC3497375

[11] Elsamee MOA Ibrahim MR Hassan MM Ashmawy ES. Leaves of Moringa, rosemary and olive as a phytogenic feed additives in muscovy duck diets. Pak J Biol Sci. (2019) 22:1–7. doi: 10.3923/pjbs.2019.1.7 30796762

[12] Zumbaugh C Murugesan G Wong E Syed B Persia M. Evaluation of a phytogenic feed additive on performance, nutrient digestion, and absorption in turkey poults. Anim Feed Sci Technol. (2020) 267:114575. doi: 10.1016/j.anifeedsci.2020.114575

[13] Malhi KK Chen J Wang T Huang M Xing K Xing H . Dietary supplementation with blended essential oils improves meat quality of broilers through SCFA-mediated gut-muscle axis. Poult Sci. (2025) 104:105911. doi: 10.1016/j.psj.2025.105911 41124995 PMC12590136

[14] Lu Y Maimaiti S Qin Z Cheng X Li J Zhou C . Effects of *Ficus carica* L. polysaccharide on the intestinal immune function and microbiota of broilers. Front Immunol. (2025) 16:1579046. doi: 10.3389/fimmu.2025.1579046 40264763 PMC12011799

[15] Shibato J Kimura A Yamashita M Shioda S Takenoya F Rakwal R. Effects of essential oil inhalation on the enhancement of plasma and liver lipid metabolism in mice. Int J Mol Sci. (2025) 26:5674. doi: 10.3390/ijms26125674 40565139 PMC12192594

[16] Gao S Wang X Bao R Yang Q Cai Q Zhang Y . Curculigo orchioides polysaccharide promotes the growth and development of Wenchang chickens via the PI3K/Akt/mTOR signaling pathway. Animals. (2025) 15:3585. doi: 10.3390/ani15243585 41463870 PMC12729267

[17] Liu W Zha P Guo L Chen Y Zhou Y. Effects of different levels of dietary chlorogenic acid supplementation on growth performance, intestinal integrity, and antioxidant status of broiler chickens at an early age. Anim Feed Sci Technol. (2023) 297:115570. doi: 10.1016/j.anifeedsci.2023.115570

[18] Ding X Giannenas I Skoufos I Wang J Zhu W. The effects of plant extracts on lipid metabolism of chickens—a review. Anim Biosci. (2023) 36:679–91. doi: 10.5713/ab.22.0272 36397703 PMC10164473

[19] Su Y Huang P Wu Z Dai W Zhang Y Zeng J. Effect of dietary supplementation with sanguinarine on meat quality and lipid metabolism of broilers. Poult Sci. (2024) 103:103925. doi: 10.1016/j.psj.2024.103925 38943809 PMC11261466

[20] Long LN Kang BJ Jiang Q Chen JS. Effects of dietary *Lycium barbarum* polysaccharides on growth performance, digestive enzyme activities, antioxidant status, and immunity of broiler chickens. Poult Sci. (2020) 99:744–51. doi: 10.1016/j.psj.2019.10.043 32029159 PMC7587896

[21] Xu D Wang X Hou X Wang X Shi W Hu Y. The effect of *Lonicerae flos* and *Rhizoma curcumae longae* extract on the intestinal development and function of broilers. Poult Sci. (2024) 103:104225. doi: 10.1016/j.psj.2024.104225 39217666 PMC11402626

[22] Yu AC Wang MA Chen L Long C Guo Y Sheng XH . Effects of dietary pretreated Chinese herbal medicine supplementation on production performance, egg quality, uterine histopathological changes, and antioxidant capacity in late-phase laying hens. Front Physiol. (2023) 14:1110301. doi: 10.3389/fphys.2023.1110301 36744028 PMC9895833

[23] Yuan J Li Y Miao J Zhang X Xiong Y Ma F . Bamboo leaf flavonoids ameliorate cyclic heat stress-induced oxidative damage in broiler liver through activation of Keap1-Nrf2 signaling pathway. Poult Sci. (2025) 104:104952. doi: 10.1016/j.psj.2025.104952 40043675 PMC11927693

[24] Sommer F Anderson JM Bharti R Raes J Rosenstiel P. The resilience of the intestinal microbiota influences health and disease. Nat Rev Microbiol. (2017) 15:630–38. doi: 10.1038/nrmicro.2017.58 28626231

[25] Oviedo-Rondón EO. Holistic view of intestinal health in poultry. Anim Feed Sci Technol. (2019) 250:1–8. doi: 10.1016/j.anifeedsci.2019.01.009

[26] Ren J Ren S Yang H Ji P. Effects of phytogenic feed additive on production performance, slaughtering performance, meat quality, and intestinal flora of white-feathered broilers. Vet Sci. (2025) 12:396. doi: 10.3390/vetsci12050396 40431489 PMC12115448

[27] Pourabedin M Guan L Zhao X. Xylo-oligosaccharides and virginiamycin differentially modulate gut microbial composition in chickens. Microbiome. (2015) 3:15. doi: 10.1186/s40168-015-0079-4 25874109 PMC4396176

[28] Zhu N Wang J Yu L Zhang Q Chen K Liu B. Modulation of growth performance and intestinal microbiota in chickens fed plant extracts or virginiamycin. Front Microbiol. (2019) 10:1333. doi: 10.3389/fmicb.2019.01333 31275268 PMC6591263

[29] Kohl KD. Diversity and function of the avian gut microbiota. J Comp Physiol B. (2012) 182:591–602. doi: 10.1007/s00360-012-0645-z 22246239

[30] Nafady AA Ghoneim SS Behour TS Akhtar M Youssef IM Abd-Elkareem M . Comparative study of gut microbiota profiles and reproductive traits in high- and low-laying chickens. Poult Sci. (2025) 104:105991. doi: 10.1016/j.psj.2025.105991 41202591 PMC12747197

[31] Flint HJ Scott KP Duncan SH Louis P Forano E. Microbial degradation of complex carbohydrates in the gut. Gut Microbes. (2012) 3:289–306. doi: 10.4161/gmic.19897 22572875 PMC3463488

[32] Ning Z Wang H Qin R Wei W Aireti N Guo S . Effects of *Lactobacillus plantarum*-fermented feed and postbiotics on the growth performance, digestibility, serum biochemistry, and caecal microbiota of chickens. Poult Sci. (2026) 105:106275. doi: 10.1016/j.psj.2025.106275 41442915 PMC12799937

[33] Ley RE Turnbaugh PJ Klein S Gordon JI. Microbial ecology: human gut microbes associated with obesity. Nature. (2006) 444:1022–23. doi: 10.1038/4441022a 17183309

[34] den Besten G van Eunen K Groen AK Venema K Reijngoud DJ Bakker BM. The role of short-chain fatty acids in the interplay between diet, gut microbiota, and host energy metabolism. J Lipid Res. (2013) 54:2325–40. doi: 10.1194/jlr.R036012 23821742 PMC3735932

[35] Koh A De Vadder F Kovatcheva-Datchary P Bäckhed F. From dietary fiber to host physiology: short-chain fatty acids as key bacterial metabolites. Cell. (2016) 165:1332–45. doi: 10.1016/j.cell.2016.05.041 27259147

[36] Xu L Wei Z Guo Y Guo B Cai L Yan J . Effects of dietary supplementation with fermented flaxseed meal on the growth performance, immune function, and intestinal microbiota of growing pigs. Anim Feed Sci Technol. (2024) 316:116079. doi: 10.1016/j.anifeedsci.2024.116079

[37] Sztandarski P Marchewka J Jaszczyk A Solka M Michnowska H Pogorzelski G . Research note: cecal microbiota and growth performance in Hubbard JA 757 broilers with and without outdoor access. Poult Sci. (2026) 105:106162. doi: 10.1016/j.psj.2025.106162 41352188 PMC12721252

